# Torque, but not FliL, regulates mechanosensitive flagellar motor-function

**DOI:** 10.1038/s41598-017-05521-8

**Published:** 2017-07-17

**Authors:** Ravi Chawla, Katie M. Ford, Pushkar P. Lele

**Affiliations:** Artie McFerrin Department of Chemical Engineering, Texas A&M University, College Station, TX 77843 USA

## Abstract

The stator-complex in the bacterial flagellar motor is responsible for surface-sensing. It remodels in response to perturbations in viscous loads, recruiting additional stator-units as the load increases. Here, we tested a hypothesis that the amount of torque generated by each stator-unit modulates its association with the rotor. To do this, we measured stator-binding to the rotor in mutants in which motors reportedly develop lower torque compared to wildtype motors. First, we employed a strain lacking *fliL*. Contrary to earlier reports, measurements indicated that the torque generated by motors in the *fliL* strain was similar to that in the wildtype, at high loads. In these motors, stator-binding was unchanged. Next, experiments with a paralyzed strain indicated that the stator-binding was measurably weaker when motors were unable to generate torque. An analytical model was developed that incorporated an exponential dependence of the unit’s dissociation rate on the force delivered to the rotor. The model provided accurate fits to measurements of stator-rotor binding over a wide range of loads. Based on these results, we propose that the binding of each stator-unit is enhanced by the force it develops. Furthermore, FliL does not play a significant role in motor function in *E. coli*.

## Introduction

The bacterial flagellar motor consists of a membrane-embedded stator and a transmembrane rotor. The motor undergoes structural and functional modifications in response to long-lived perturbations in the cell’s thermal^1, 2^, chemical^3–7^, electrical^8, 9^, and mechanical^10–14^ environments. Structural-remodeling likely improves the odds of survival. For example, the remodeling of rotor-complexes enables an adaptation that extends the range of signal detection and improves the ability to respond to chemical signals^5, 7^. In *Escherichia coli*, the motor is rotated by a set of stator units consisting of two proteins, MotA and MotB, that utilize the proton motive force (pmf) to deliver a torque to the rotor^15^. In previous work, we showed that the stators are mechanosensitive and enable motor-adaptation in response to dramatic increases in viscous loads^10^. This adaptation is facilitated by stator-remodeling, which aids the motor recruit additional stator-units under high loads^10, 11, 13^. Stator-remodeling modulates torque, which is likely to be advantageous for swimmers that find themselves in high-viscosity environments or for swarmers that need to overcome higher shear when swarming on substrates. The ability of the motor to sense and adapt to mechanical signals makes it one of the clearest examples of a mechanosensitive, biological motor^10, 16^.

The role of the motor in surface-sensing and in triggering of biochemical pathways is of significant interest^17–20^. However, the mechanisms underlying its response to mechanical signals remain unclear. Previously, we proposed a mechanism for remodeling that was torque-dependent^10^. According to our hypothesis, the torque generated by individual stator-units modulates the accessibility of cryptic binding sites, thereby controlling the binding-affinity to the rotor. At high loads, a single stator-unit generates a significant amount of torque (~100–200 pN-nm), likely exposing those binding sites. Hence, in motors that experience significant loads, the stator is expected to remodel and recruit additional units.

To test the hypothesis, we measured stator-binding in mutants in which motors have been reported to develop low torques under high loads. Recent reports suggest that in *E. coli* and *S. enterica*, flagellar motors cannot generate high torques in the absence of FliL^21^ (a membrane-associated 17 kDa protein^22^), resulting in the inhibition of swarming. However, these findings are at odds with earlier reports by the Macnab group that suggested that FliL does not regulate chemotaxis and motility in *E. coli* ^23^. Nonetheless, several other bacterial species have been observed to be deficient in swimming and swarming when lacking *fliL*; presumably, because the motors fail to deliver adequate torque^24–26^. Hence, we measured torque and stator-binding in a strain of *E. coli* lacking *fliL*. Our measurements indicated no significant degradation in motor-torque over a wide-range of loads. Consistent with our hypothesis, stator-rotor association was not affected either. Furthermore, the mutant swarmed, in agreement with recent reports^27^. Next, we worked with a strain carrying paralyzed stators that were unable to develop torque, as a result of which, tethered cells remained predominantly locked. In this strain, stators exhibited weaker association with the rotor lending support to the notion that remodeling is regulated by absolute torque. An analytical model was developed that explained load-dependent stator-remodeling by incorporating an exponential dependence of the stator-unit’s off-rate constant on the amount of force delivered to the rotor. The model was able to accurately fit the dependence of stator-binding on viscous loads measured by Tipping and co-workers^11^. Our results suggest that a torque-dependent mechanism likely underlies mechanosensitive remodeling of the motor and emphasize the indispensable role of remodeling in swarming.

## Results

### Torque and FliL at High Loads

**Figure 1:**
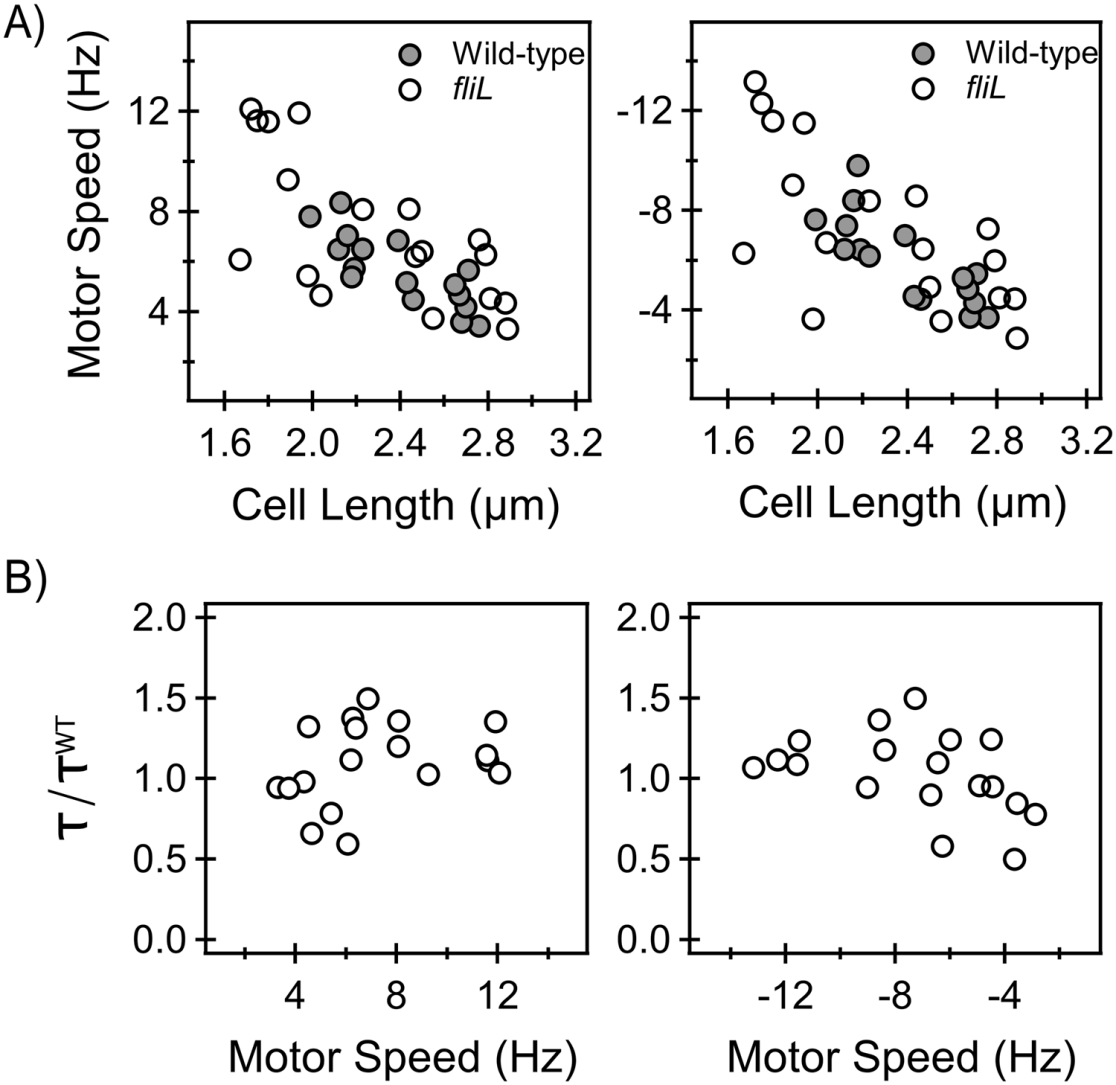
(**A**) *Steady*-*state speeds versus lengths:* The rotational speeds and cell lengths in the *fliL* (open circles, n = 18 motors) and wild-type (filled circles, n = 16 motors) strains are indicated. The left panel indicates speeds in the CCW direction and the right panel indicates speeds in the CW direction. The mean CCW speeds were 5.65 ± 1.44 Hz (wildtype strain) and 7.24 ± 2.93 Hz (*fliL* strain). The mean CW speeds were 5.96 ± 1.75 Hz (wildtype strain) and 7.28 ± 3.19 Hz (*fliL* strain). Differences in the mean speeds in the two strains were not significant (p-value > 0.05). (**B)** *Torque versus speed*: The torque generated by individual motors in the *fliL* strain was calculated from rotational speed and cell geometry. The left and right panels indicate the CCW and CW data, respectively. *τ* represents the torque generated by individual motors in the *fliL* strain and *τ* ^*WT*^ represents the mean torque generated by motors in the wildtype strain.

Employing the tethered cell assay, we determined the torque developed by individual motors at near-stall viscous loads in the *fliL* and wildtype strains. Cells were tethered to the glass surface via their filaments and were observed to rotate at constant speeds several minutes after tethering (*Materials and Methods*). The cells switched between clockwise (CW) and counterclockwise (CCW) directions of rotation. A subset of the total data that had a narrow distribution of cell lengths was selected for further analysis (mean cell length = 2.28 ± 0.44 μm, *fliL* strain and mean cell length = 2.40 ± 0.26 μm, wildtype strain). The rotational speeds of cells belonging to the *fliL* strain are shown in Fig. 1A, for both directions. The corresponding wildtype speeds are also indicated. The flagellar motor generates nearly constant torque at high loads and as a consequence, its speed decreases with increasing load^28^. Consistent with this, speeds were observed to decrease with increasing cell lengths (Fig. 1A). The motors in the two strains exhibited similar speed versus cell-length characteristics in either direction and the difference in the mean motor speeds was not significant (p-value > 0.05, Fig. 1A). To compare the torque generated in the two strains, we first calculated the torque developed by each motor in the *fliL* strain by taking into account the size of the cell (see *Materials and Methods*). Individual torque was then normalized by the mean torque generated by wildtype motors. The normalized torque over a range of speeds is indicated in Fig. 1B, for both directions. The mean normalized values were 1.09 ± 0.06 (CCW) and 1.03 ± 0.06 (CW). We concluded from these results that the absence of FliL did not adversely affect the torque developed by flagellar motors at near-stall loads.

### Torque and Stator-Rotor Association

#### *fliL* strains

**Figure 2:**
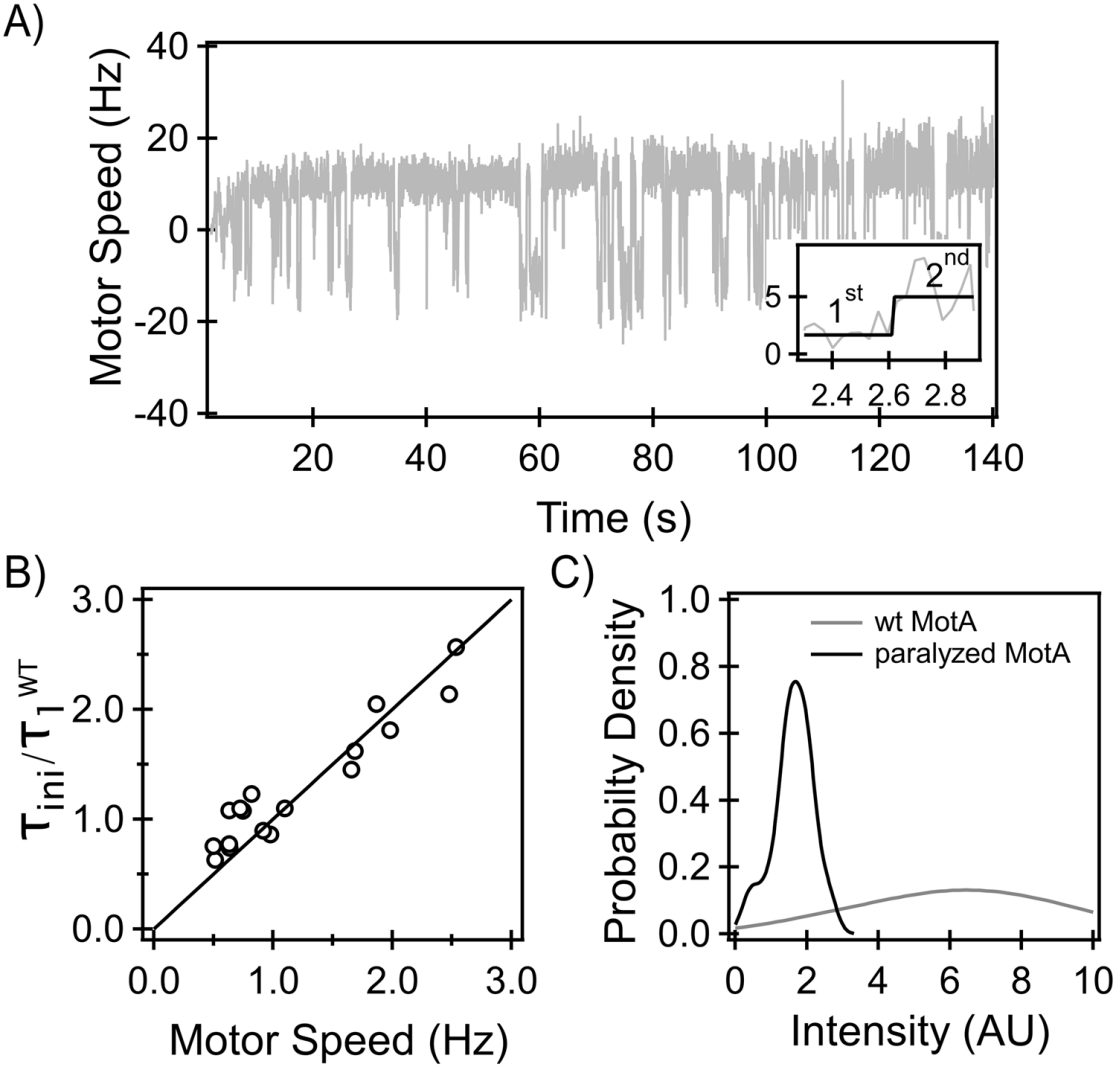
(**A**) *Response to mechanical stimulus:* The adaptation in response to a mechanical stimulus in a representative *fliL* motor is visible from the step-wise increments in speed over time. The inset shows the response of the motor (gray) over the first few seconds and the black line is the output of the step-finding algorithm, indicating the 1^st^ and the 2^nd^ step. (**B)** *Stator-binding:* The initial torque developed by *fliL* motors (*τ*_*ini*_), normalized by the mean torque developed by single stator-units (*τ* ^*WT*^_*1*_) in wildtype motors, is indicated. *τ*_*ini*_ was determined from the cell geometry and the initial speed (inset Fig. 2A). The data near the lower left corner corresponds to 1 unit (*τ*/*τ* ^*WT*^_*1*_~1) and the data near the upper right corner represents two units (*τ*/*τ* ^*WT*^_*1*_~2). (**C**) *Stator-binding in paralyzed motors:* Kernel density estimates of stator-intensities in paralyzed motors (black) and functional motors (gray) are shown. The mean intensities were 1.5 ± 0.14 a.u. (paralyzed) and 4.63 ± 0.4 a.u. (wildtype). The difference in the means was highly significant (p-value < 0.01). The raw data has been included in the supplementary text (Fig. S1).

The rotational speed (Fig. 1A) depends not only on the torque developed by each stator-unit but also on the number of units bound to the rotor. To determine the number of bound stator-units, we applied step increments in viscous loads on individual motors by allowing cells to tether. Cell rotation was recorded immediately following the onset of tethering^10^. The response of a representative *fliL* motor is shown in Fig. 2A. The motor initially rotated at a low speed. Over the next hundred seconds, the speed increased in a step-wise fashion to reach a maximum value (~12.75 Hz). The adaptation in speed is consistent with our observations with wild-type motors (data not shown) and is indicative of stator-remodeling. This confirmed our previous result that flagellar mechanosensing is independent of FliL^10^.

At very low loads, stators deliver a small amount of torque (~10 pN-nm) and the number of bound units at these loads has been observed to be ~1–2 units in wildtype motors^10^. To estimate the number of units in *fliL* motors at very low loads, we employed a step-finding algorithm and calculated the mean speed at each step in the speed data^10^. Next, we identified the initial speeds at which the *fliL* motors rotated (indicated by the 1^st^ step in the inset in Fig. 2A). Based on these speeds and the cell geometry, the initial torque generated (*τ*_*ini*_) was determined for each motor. The torque values were normalized by the mean torque generated by single-units in wildtype cells (*τ* ^*WT*^_*1*_). The normalized torque data (n = 17 motors) are shown in Fig. 2B as a function of speed and can be approximately grouped into two. The first group on the lower left corner represents motors that carried single units initially (*τ*_*ini*_/*τ* ^*WT*^_*1*_~1) and the second group, on the upper right corner, represents motors that carried two units (*τ*_*ini*_ / *τ* ^*WT*^_*1*_~2). Since the load on the motor prior to tethering is very low, this indicated that motors in *fliL* mutants employ no more than 1-2 stator-units at very low loads, similar to the wildtype cells^10^.

To measure stator-rotor association at high loads, we obtained the mean step-increment in speed for each motor from the adaptation data. Dividing the final speed attained by the motor by the step-size enabled us to estimate the number of units bound to each rotor upon the completion of remodeling. This approach is appropriate since, at high loads, each new unit that binds to the motor has been shown to contribute equivalent torque as its predecessors^29^. Calculations indicated that the mean number of stator-units engaged by the motors at high loads in the *fliL* mutant was 8.7 ± 3.0, similar to the wildtype (9.1 ± 1.87). Thus, the lack of FliL had little effect on the stator-rotor association.

#### Paralyzed motors

To test whether a full complement of stators could assemble around the rotor when the torque is degraded, we employed a strain that carried chromosomal *eyfp-motB* and a point mutation in *motA* that resulted in paralyzed motors^30^. In this strain, the paralyzed stator-units bind to the rotor but cannot generate a torque causing the tethered cell to remain predominantly locked. Cells were tethered for ~ 600 seconds and fluorescently-labeled stators associated with tethered motors were imaged via TIRF. The intensities of the motor-bound stator-proteins were quantitatively determined using previous protocols^6^. The intensities in the paralyzed and the wildtype strain are indicated in Fig. 2C. The difference in the mean intensities was highly significant (p-value < 0.01, Fig. 2C). Evidently, functional units were able to bind to the motor in higher numbers relative to the paralyzed units. The mean intensity in wildtype motors was ~3 times higher than the mean intensity in paralyzed motors. A functioning stator-unit generates a significant amount of torque (~100–200 pN-nm) in a tethered cell. On the other hand, in paralyzed strains the stators do not generate a torque. Therefore, these results are consistent with the hypothesis that torque regulates binding of the stator-units to the rotor.

### Torque and Swarming

#### Torque and FliL over a range of load

**Figure 3:**
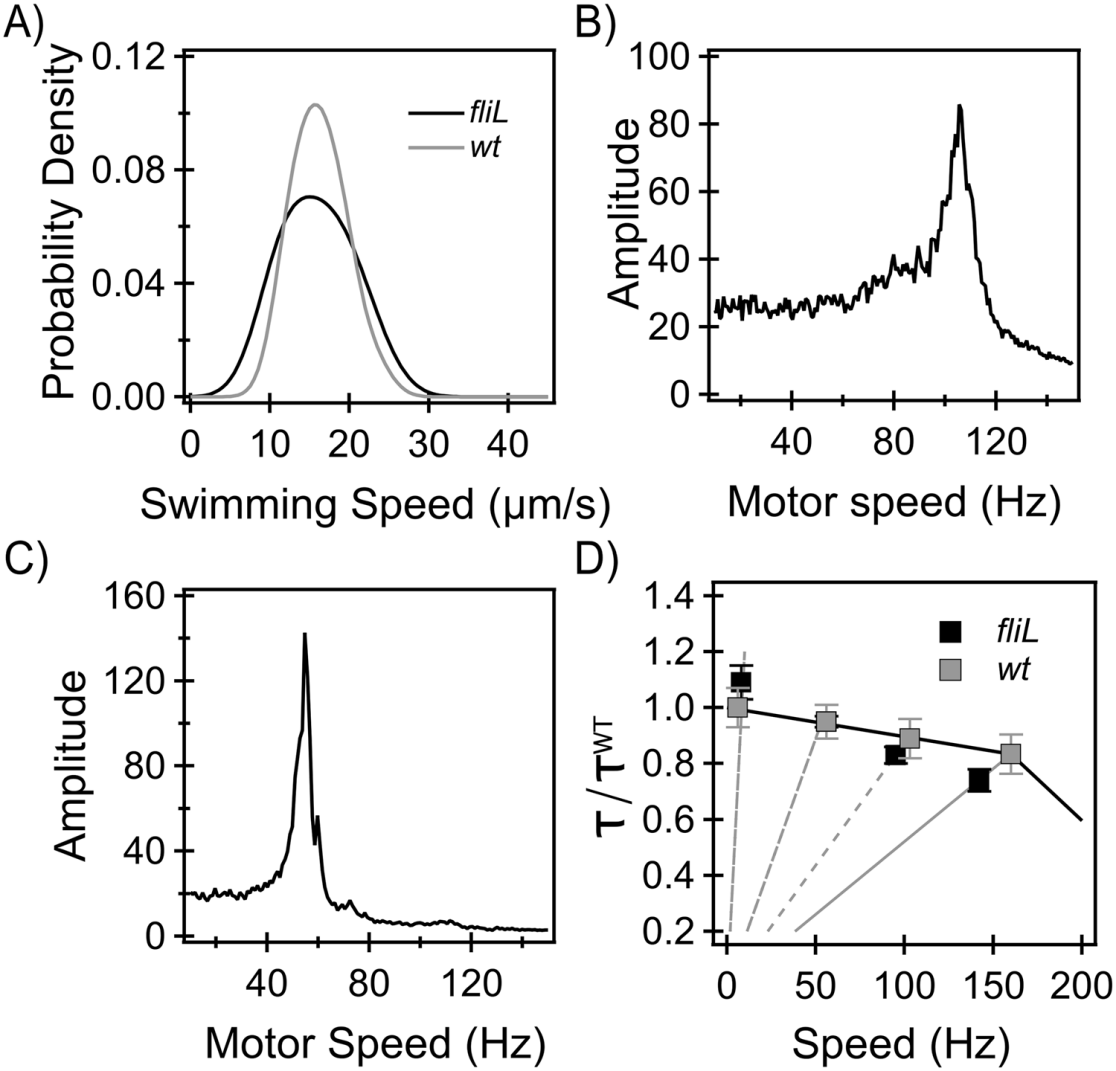
(**A**) *Swimming speeds*: Kernel density estimates of swimming speeds in *fliL* (black) and wildtype (gray) strains are shown. The difference in the mean swimming speeds was significant (p-value < 0.05). The mean speed in the wildtype strain was 20% higher than that in the *fliL* strain. (**B)** and (**C)** *Tethered beads:* Signals from representative *fliL* motors, one rotating a 750 nm and another rotating a 1 μm bead. Peaks correspond to ~106 Hz and ~55 Hz, respectively. (**D)** *Torque-speed curve:* The average torque generated by motors in the *fliL* strain (black symbols) and in the wildtype strain (gray symbols) is plotted against the average motor speed. The torque (*τ*) has been normalized by the maximum torque (*τ* ^*WT*^) measured in wildtype tethered cells.

To determine if FliL affected motor-function at viscous loads other than near-stall conditions, we measured torque in *fliL* strains over a range of loads. Due to hydrodynamic screening near solid substrates, the viscous loads (*ζ*) on motors in swarmer cells are likely higher than the viscous loads on motors in swimmer cells. Hence, we first tested motor-performance at swimming loads (*ζ*_*swimming*_). Wildtype motors at these loads typically rotate at ~165–175 Hz^31^. To determine if the *fliL* mutants exhibited subnormal motor torques at *ζ*_*swimming*_, we compared swimming speeds in wild-type and *fliL* strains in liquid growth media. The motion of swimming cells was recorded and speeds were quantitatively determined as discussed in *Methods and Materials*. The distributions of speeds obtained from our measurements are shown in Fig. 3A for the wildtype and *fliL* strains. The difference in the mean swimming speeds was significant (p-value < 0.05, Fig. 3A). Assuming a similar number of flagella per cell in the *fliL* and the wildtype strains, the torque generated by motors in the *fliL* strain was estimated to be ~90% of the torque generated by wildtype motors.

Motors were subjected to higher loads by tethering them with beads of different sizes. Single-motor rotation was measured with a photomultiplier setup that enabled high-speed tracking of bead-rotation^32^. The photomultiplier-output signals were sinusoidal, each wave corresponding to one full turn of the motor. Power spectral densities (PSD) were calculated from the output signals to obtain the rotational speed. Representative PSDs for a *fliL* motor rotating a 750 nm bead and another motor rotating a 1 μm bead are shown in Fig. 3B and C, respectively. The peaks were sharp, indicating a good signal-to-noise ratio, enabling us to accurately determine the frequency of rotation. For wildtype motors, our measurements yielded mean speeds of ~56 ± 6 Hz (1 μm, 31 motors) and ~105 ± 24 Hz (750 nm, 42 motors), similar to earlier measurements with the wildtype strain^28^. The mean speeds for the *fliL* strain were ~56 ± 17 (1 μm, 33 motors) and ~95 ± 14 Hz (750 nm, 30 motors). The differences in the means between the two strains were insignificant (p-value > 0.05).

The average torque was then determined for all the viscous loads used in this work (*Materials and Methods*). A comparison of the motor torque vs speed curve for the *fliL* and wildtype strains is shown in Fig. 3D. The differences between the two curves were minor, suggesting that the absence of FliL does not significantly affect torque-generation. Consequently, stator-rotor association is unlikely to be affected either.

### Swarming

**Figure 4:**
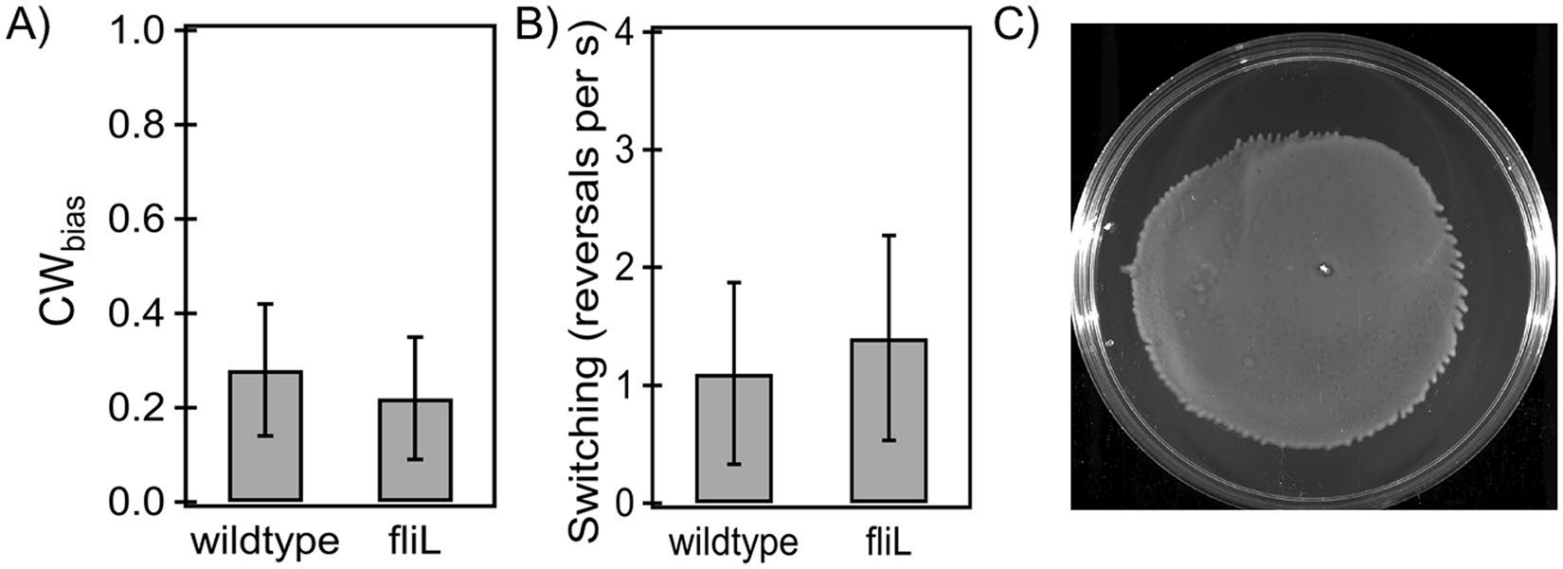
(**A**) *Probability of CW rotation*. The mean CW_bias_ was 0.28 ± 0.03 (n = 16 motors) in the wildtype strain and 0.22 ± 0.03 (n = 18 motors) in the *fliL* strain. The difference in the means was not significant (p-value > 0.05). (**B)** *Switching-frequencies*: The mean reversal frequency was 1.1 ± 0.77 per second in the wildtype strain and 1.4 ± 0.87 per second in the *fliL* strain. The difference in the means was not significant (p-value > 0.05). (**C)** *Swarming*: A representative swarm-plate inoculated with the *fliL* strain. The average swarm diameter was 4.06 ± 1.13 cm, n = 10 plates (8-hour incubation).

Motor-reversal (switching) is indispensable for swarming^33^. Therefore, we carried out measurements to test whether FliL had a measurable impact on switching. This was done by determining the CW_bias_ (probability of CW-rotation) and motor-reversal frequencies. As indicated in Fig. 4A, the mean CW_bias_ was not significantly different between the wildtype and *fliL* strains (p-value > 0.05). The difference between the mean reversal frequencies was not significant either (p-value > 0.05).

Considering that neither torque nor switching were inhibited in the *fliL* strain, we tested whether the loss of FliL indeed prevented *E. coli* from swarming. Figure 4C shows a representative image of a swarm plate inoculated with the *fliL* strain in an environmental chamber (*Methods and Materials*). As evident, the loss of FliL did not prevent swarming. This was further confirmed by imaging swarm-motion under phase microscopy with a 40x, ph3 objective. The results were reproducible (swarm diameter = 4.06 ± 1.13 cm over 8 hour incubation, 10 plates).

### Model for torque-dependent stator-remodeling

Stator-units that are a part of a functioning stator-complex have been shown to continually exchange with freely-diffusing units in the cell-membrane^34^. According to our hypothesis, the number of stator-units associated with each motor varies with the amount of torque developed by each unit. To explain the dependence of stator-binding on torque, we assumed that the dissociation rate-constant (*k*_*off*_) is a function of the force applied by each unit on the rotor, *F*, whereas the on-rate (*k*_*on*_) remains unchanged. The stator-exchange kinetics are represented as follows:

1 $$\frac{dn}{dt}={k}_{on}UB-{k}_{off}(F)n\,$$

here, *n* represents the number of bound units (or occupied sites) and B represents the number of vacant binding sites. *U* is the total number of units available in the cell that remains more or less constant. At steady state (ss), the total available binding sites B_T_ (=*B*_*ss*_ + *n*_*ss*_) and the number of bound units are related by:

2 $$\frac{{n}_{ss}}{{B}_{T}}=\frac{1}{1+{k}_{off}(F)/{k}_{on}U}$$

We assumed that *k*_*off*_ depends exponentially on force, similar to the Bell model for slip-bonds^35^, although with a negative sign in the exponent, such that *k*_*off*_ = *k*_*off*_^*0*^ *exp*(−*Fδ/k*_*B*_ *T*). Here *k*_*off*_^*0*^ is the off-rate constant at zero force and *δ* is the characteristic bond length.

3 $$\frac{{n}_{ss}}{{B}_{T}}=\frac{1}{1+\phi exp(-F\delta /{k}_{B}T)}$$

where $\phi =({{k}_{off}}^{0}/{k}_{on}U)$. We constrained $1/\phi$ ~ 0.01–1 based on prior estimates with other motor-associated proteins^6^. The amount of force generated by each unit, *F*, is known to increase with load and was assumed to linearly decrease with rotational speed.

4 $$F={F}_{max}(1-\frac{v}{{v}_{max}})$$

*F*_*max*_ is the maximum force (at near-stall conditions) that a single unit can apply on a rotor with a radius *r* = 22.5 nm. More accurate, non-linear models for the dependence of force on load are available elsewhere^36, 37^. From prior measurements, *v*_*max*_ = 300 Hz^38^. Also, *F*_*max*_ = *τ*_*max*_/*(n*_*ss*_ *r)* and *τ*_*max*_ is the total stall-torque applied by a full complement of stator-units^29^. The dependence of motor-speed on viscous loads (*τ* = *ζν*) was estimated from the experimentally-measured torque-speed relationships^28, 29^.

**Figure 5:**
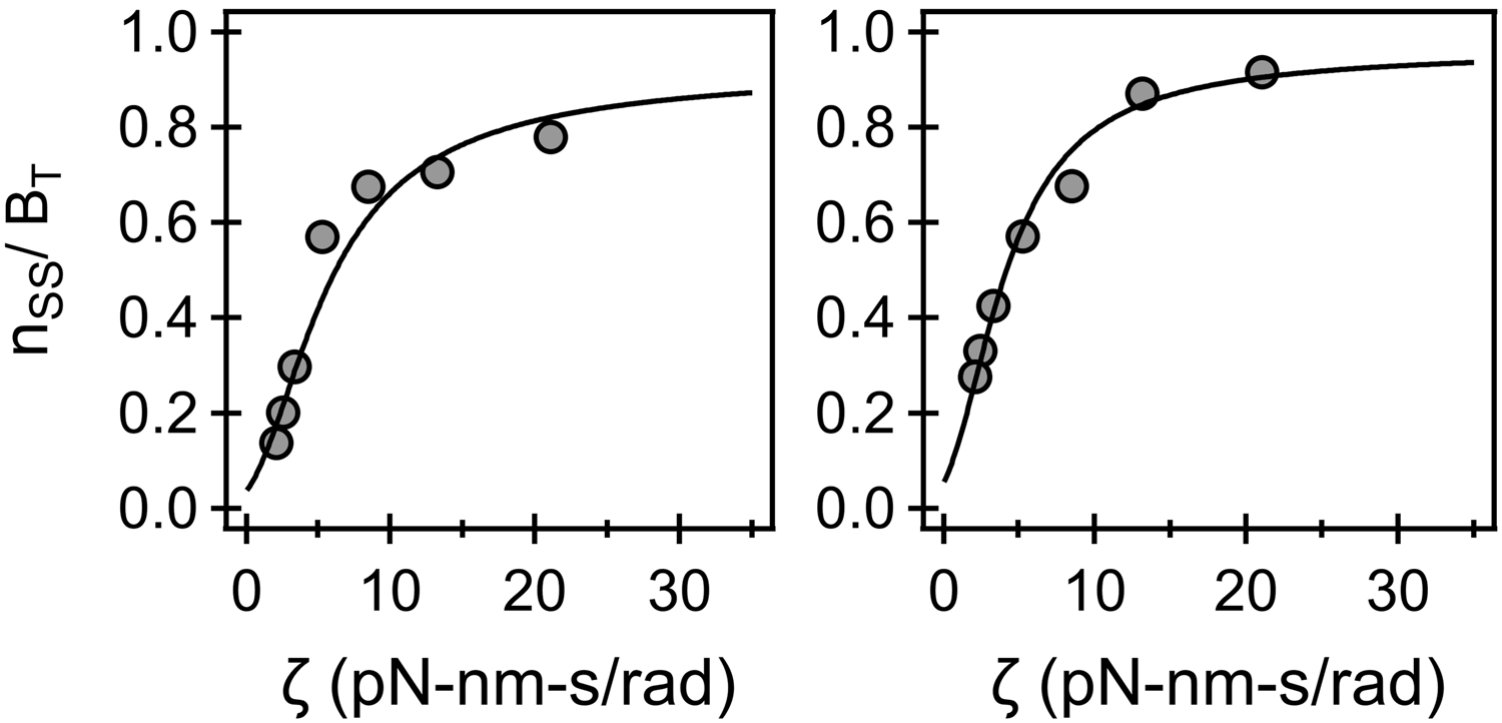
*Analytical fit to experimental data*. The measurements of stator-binding to motors over a range of loads are indicated by symbols for the CCW (left-panel) and the CW (right-panel) directions^11^. The model-fit is indicated by the curve. For the CCW direction, 1/φ~0.053 and δ = 2.48 nm and for the CW direction, 1/φ~0.035 and δ = 2.65 nm. The values of the coefficient of determination (R^2^) were 0.95 (CCW) and 0.99 (CW), see supplementary text for additional details.

Equations 3 and 4 were combined to yield a single analytical expression that was fitted to the measurements of stator-rotor binding over a range of viscous loads^11^. The fits to the experimental data for the CCW and CW directions are indicated in Fig. 5. As detailed in the supplementary text, the model was linearized and parameter values were obtained via linear regression. The fits were excellent (Fig. 5) and yielded characteristic lengths of *δ*~2.48 nm (CCW) and *δ*~2.65 nm (CW). These lengths are approximately half the size of a single step of the rotor and provide a measure of the distance over which force F acts during stepping to modulate binding. Thus, the simple model provided excellent fits and matched the shape of the non-linear experimental data reasonably well, providing further support to the torque-hypothesis.

## Discussions

Several studies have shown that the motor is highly dynamic - it remodels component-complexes to adapt its function in response to a variety of stimuli^1–14^. The stator-complex is the mechanosensitive part of the flagellar motor and it responds to mechanical stimuli by remodeling. A sudden and significant increase in the load on a motor that is rotated by a single unit reduces the flux of protons through the unit as well as the rotational speed. Simultaneously, the stator-unit increases the amount of force delivered from <1 pN to ~5–15 pN, depending on the load. It is less likely that a change in the flux of protons or the drop in rotational speeds is the signal for remodeling. This is because a disruption in the pmf, which reduces speeds as well as flux, has been observed to result in the disassembly rather than the assembly of stator-units at high loads^8, 9^.

In this work, we have tested the notion that torque regulates stator-binding to the rotor^10^. It is likely that each stator-unit carries cryptic binding sites that typically remain inaccessible when the unit delivers a low torque (at very low loads). However, when the unit delivers a high torque (at high loads), these sites become accessible due to the development of high tensile forces during a power-stroke. In such a mechanism, higher torque strengthens the stator-rotor association leading to stator-remodeling under high loads. We tested this idea by measuring stator-binding in mutants in which torque was anticipated to be weak or negligible at high loads. Our results were consistent with this hypothesis and indicated that stator-binding increased with the amount of torque generated. An analytical model was developed that incorporated an exponential dependence of the stator-unit’s dissociation rate-constant on the force generated by that unit. The model provided accurate fits to the available experimental data, and suggested a characteristic bond-length (*δ*) that was ~half the size of a rotor-step. It would be interesting to determine in the future if there is a correspondence between *δ* and the nature of single-steps in the motor. Specifically, does each unit combine the action of two half-steps to complete one full step on the rotor?

In principle, the mechanism underlying the stator’s response to mechanical stimuli can explain the motor *resurrection* that was observed with the paralyzed strain previously^30^. In those experiments, cells belonging to the paralyzed strain were tethered and the expression of functional stator-units was induced from a plasmid. Following induction, each newly-expressed functional stator-unit that bound to the motor experienced a significant viscous load that modulated the torque it delivered, thereby stabilizing its association with the rotor. This resulted in a step-wise increment in speed similar to the data in Fig. 2A.

In *Vibrio alignolyticus*, experiments suggest that FliL localizes with stators in the sodium-driven polar flagellum^39^. Defects in FliL result in the degradation of swimming and swarming in *Proteus mirabilis* ^26^. However, in *E. coli* earlier reports suggested that FliL is not important in swimming and chemotaxis^23^. Our quantitative measurements confirmed the earlier findings and ruled out a role for FliL in any of the important motor-related functions including torque-generation, switching and mechanosensitive remodeling at high loads. To exclude the likelihood that our observations were specific to the strains developed in our lab, we tested motor responses to high loads and swarming in *fliL* mutants (JP1297 and JP633a) obtained from the Harshey group^21^. Measurements revealed no significant differences between the mean rotation-speeds in the JP1297 and wildtype strains (Fig. S2, supplementary text). In our hands, the JP633a strain was able to swarm.

## Conclusions

In summary, we have experimentally tested a probable mechanism for the remodeling of stators in flagellar motors in response to mechanical stimuli. Our findings are consistent with the notion that higher torque exposes cryptic binding sites that strengthen stator-binding to the rotor. An analytical model that incorporated an exponential dependence of the stator-unit’s off-rates on the torque delivered was able to fit previously measured load-binding curves accurately. The combined experimental and analytical results presented here represent the first steps towards establishing a plausible mechanism for stator-mechanosensitivity and motor-adaptation.

## Materials and Methods

### Strains and media

The *fliL* strain (PL111) was generated by employing the λ-red mediated homologous recombination technique to delete nt 61–405 in the *fliL* gene^40^. The deletion was confirmed by sequencing. Mutations in *fliL* were previously observed to result in intergenic mutations in the *motB* region of *Rhodobacter sphaeroides* ^25^. Sequencing revealed no such compensatory *motB* mutations in our strain. The paralyzed *motA* strain (PL64) was generated by exchanging the wildtype *motA* allele with a motA mutant (parent strain HCB84), in a strain carrying genomic *eyfp-motB* ^10^. Strains JP1297 (∆*fliL*, carrying the sticky *fliC* allele) and JP633a (∆*fliL*) were obtained from the Harshey group^21^. Overnight cultures were grown from isolated colonies in 5 mL of Tryptone broth (TB) and day cultures were subsequently grown (1:100 dilution) in 10 ml of fresh TB at 33 °C. Swarm-agar plates (Peptone, 10 g/L; NaCl, 5 g/L; Beef extract, 3 g/L; 0.45% Eiken Agar, 0.5% Glucose) were prepared fresh, dried after pouring for an hour at room temperature and then inoculated with the strains of interest (2 μl, overnight culture grown at 30 °C).

### Motility Assays

#### Tethered cells

Cells were prepared for motor-assays as discussed elsewhere^32^. Briefly, cells were grown to an OD_600_~0.5 and then washed several times in motility buffer (0.01 M Phosphate buffer, 0.067 M NaCl, 10^−4^ M EDTA, 0.01 M Sodium Lactate and 1  μM Methionine, pH~7.0). Standard glass flow cells were prepared by using double-sided adhesive tapes to stick two glass surfaces together. Cells were sheared and tethered via a sticky filament mutant that adheres to glass and beads^41^. Cell-rotation was imaged and recorded on a Nikon microscope (Nikon Eclipse Ti-E) with a 20x phase objective at ~60 fps with a CCD camera (DCC1545M-GL, Thorlabs Inc). Bead-rotation was imaged on a Nikon Optiphot with a 60X phase objective coupled to a photomultiplier setup^32^. *Swarming:* Swarm-assays were carried out in an environmental chamber (ETS Model 5472, Electro-Tech Systems, Inc) that allowed a fine control over humidity and temperature. Swarm-plates were imaged ~8 hours after inoculation with a digital camera (Nikon Coolpix L330). *Swimming*: Cells were grown to an OD_600_~0.5 in TB and then diluted in fresh TB (1:40 dilution). The dilute suspension was observed in a standard flowcell and cell-motion was recorded with the Thorlabs camera at 60 fps.

### Data Analysis

#### Tethered cells

Videos of tethered cells were analyzed with custom-written codes in MATLAB to determine the angular speed as a function of time^42^. Mean speeds for individual cells were determined from Gaussian fits to speed-distributions. *Swarming:* ImageJ plugins were employed to determine swarm radii from swarm images following previous protocols^43^. Briefly, since swarms rarely progressed symmetrically, the swarm expanse was first determined by manually drawing swarm-boundaries around the colony. Swarm-radius was then reported as the radius of an equivalent circular area corresponding to the selected region. *Swimming:* Most cells swam in straight lines for limited time-periods in the liquid medium. For each cell, the frames over which straight-line motion was observed were averaged which resulted in a single image with bright streaks on a gray background. The corresponding length of the straight-line intensity profile was determined and divided by the period of observation to obtain swimming speed.

### Torque Calculations

Cell-tracking enabled quantitative estimates of cell-geometries and the drag on tethered-cells was determined based on previous approaches^44^. A 2 μm long and 1 μm wide cell body tethered at a distance of 0.75–1 μm from the center offers an effective viscous load of ~150 pN-nm-s/revolution. The drag coefficient of a bead tethered to a filament stub and undergoing rotation along a circular trajectory with a non-zero eccentricity can be determined by representing the stub as a thin ellipsoid^29^. For stub lengths (~0.1–0.4 μm) and eccentricities (~0.15–0.5 μm), the loads due to 750 nm and 1000 nm beads at room temperature are ~9 and ~20 pN-nm-s/revolution, respectively.

### Statistical analysis

All statistical analyses were performed in MATLAB either with the Student’s t-test or the non-parametric Wilcoxon rank-sum test. Results with p < 0.05 were considered statistically significant and p < 0.01 were considered highly significant.

## Electronic supplementary material

Supplementary Information
