# Supplementary material for: Torque, but not FliL, regulates mechanosensitive flagellar motor-function
Source: Sci Rep. 2017 Jul 17;7:5565. doi: 10.1038/s41598-017-05521-8 (PMC5514156; doi:10.1038/s41598-017-05521-8)
Supplement: Supplementary file 1 — Supplementary Information [file 41598_2017_5521_MOESM1_ESM.pdf]

Supplementary Information

Title: Torque, but not FliL, regulates mechanosensitive flagellar motor-function

Authors: Ravi Chawla, Katie M. Ford, Pushkar P. Lele*

Affiliation: Artie McFerrin Department of Chemical Engineering,

Texas A&M University, College Station, TX-77843

***Corresponding author:** plele@tamu.edu

Stator-Rotor Binding

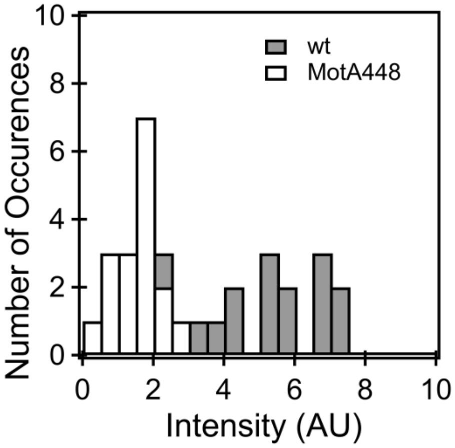

Figure S1: Raw intensity data from figure 2C. Differences between the two means were statistically significant, with fewer eYFP-MotB bound to individual rotors in paralyzed motors.

Steady-state speed

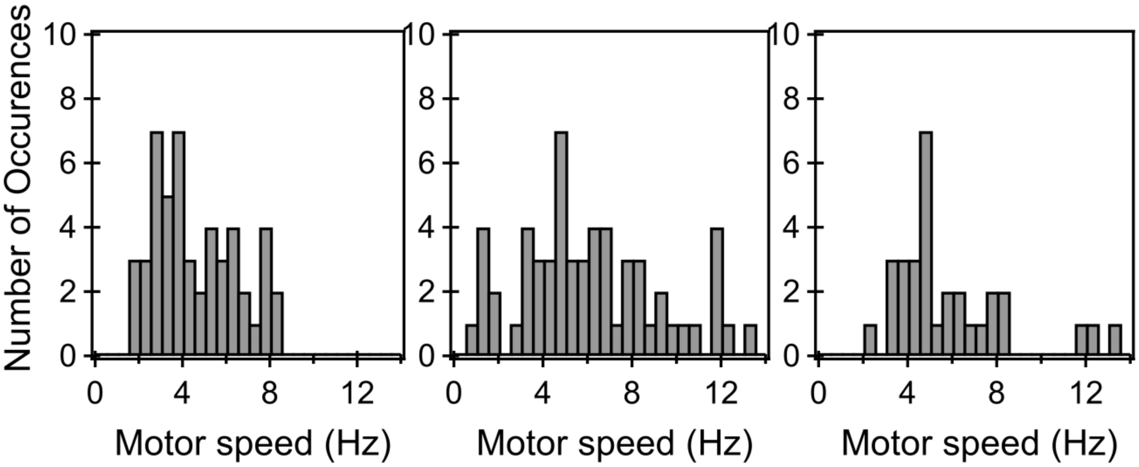

Figure S2. S*peed comparisons*. Steady-state speed distributions determined for the wild-type motors (left panel, MT02, 50 motors), *fliL* mutant (middle panel, strain PL62, 58 motors) and another *fliL* mutant (right panel, strain JP1297; n=30 motors) are shown. The p-values were > 0.05.

Model-linearization and best-fits

We linearized the model presented in equations 3 and 4 as:

$$\log\left(\frac{B_T}{n_{ss}} - 1\right) = \log(\varphi) - {\tau^1}_{max}\delta\left(1 - \frac{\tau}{V_{max}\zeta}\right)/rk_BT \quad (1)$$

The experimental data was then replotted, $\log\left(\frac{B_T}{n_{ss}} - 1\right)$ vs τ/ζ. The linear fits are indicated in Fig S3.

The R^2 values were 0.95 (CCW) and 0.99 (CW).

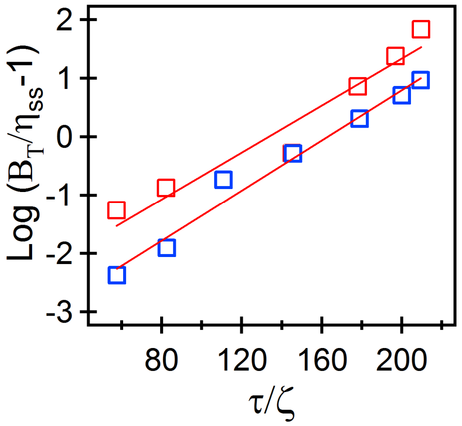

Fig S3. *Linear fit.* The blue and red symbols indicate the CW and CCW data, respectively and the continuous lines indicate fits.
